# Supplementary figures and images for: Polyploidy of semi-cloned embryos generated from parthenogenetic haploid embryonic stem cells
Source: PLoS One. 2020 Sep 10;15(9):e0233072. doi: 10.1371/journal.pone.0233072 (PMC7482839; doi:10.1371/journal.pone.0233072)

**A**

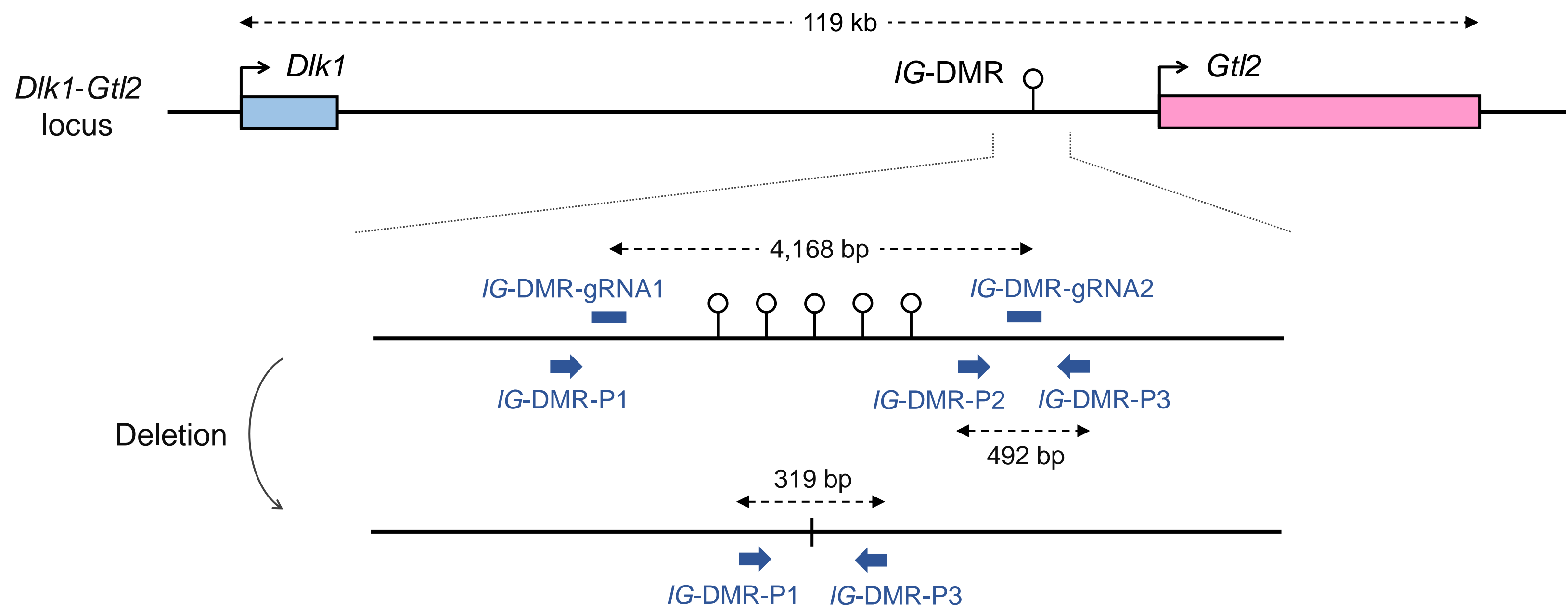

**B**

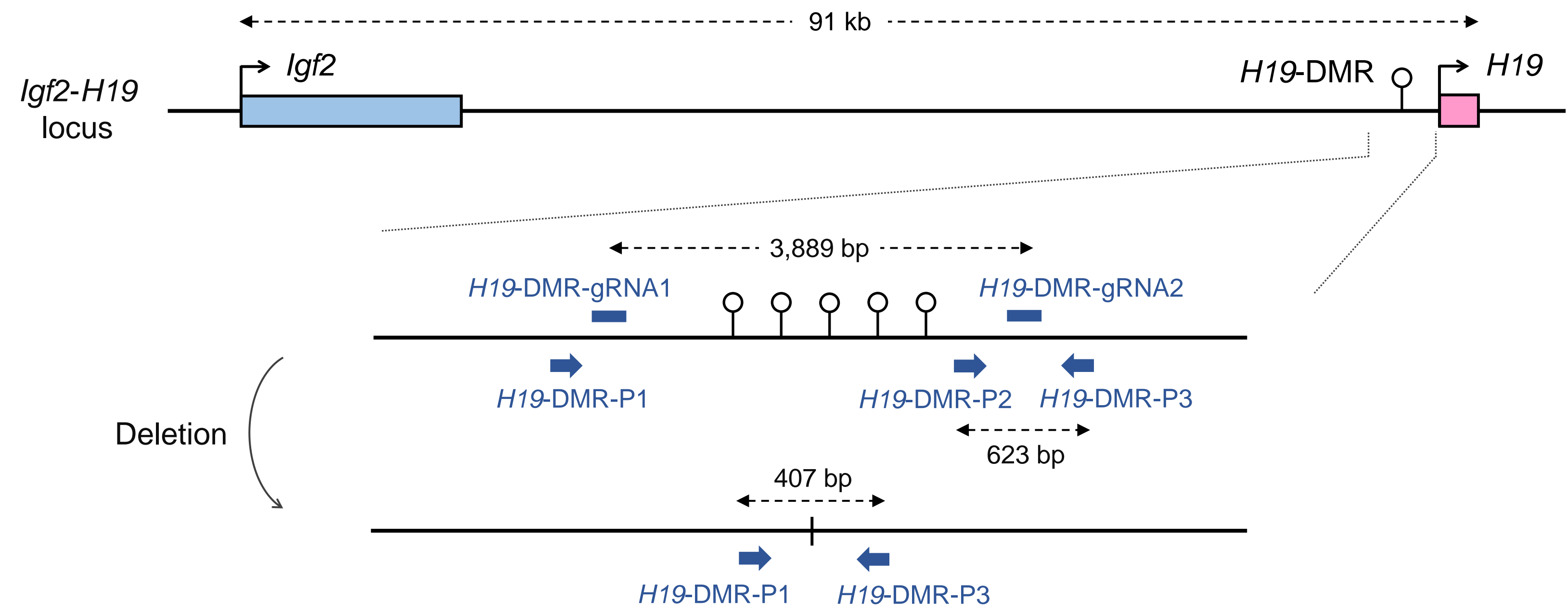

**C**

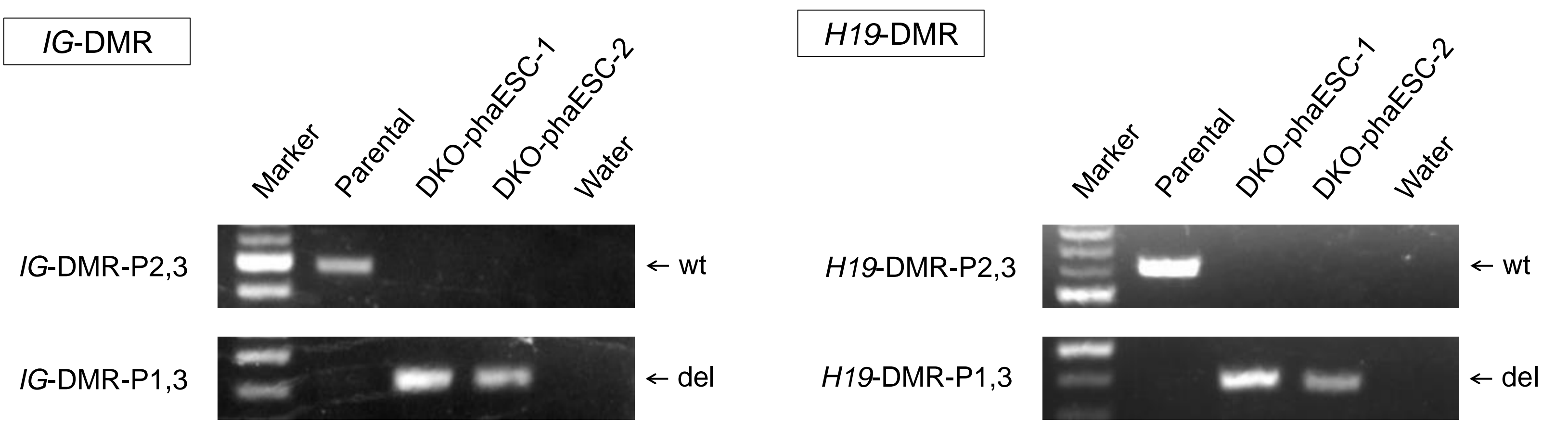

Supplement: S1 Fig — (A) A design of gRNAs and primers targeting the deletions of the IG-DMR. (B) A design of gRNAs and primers targeting the deletions of the H19-DMR. (C) PCR fragments flanking both IG-DMR (319 bp) and H19-DMR (407 bp) by primers targeting deleted loci were observed in 2 DKO-phaESC lines, whereas the deleted sequences were absent in DKO-phaESC-1 and DKO-phaESC-2. (PDF) [file pone.0233072.s001.pdf]

**A**

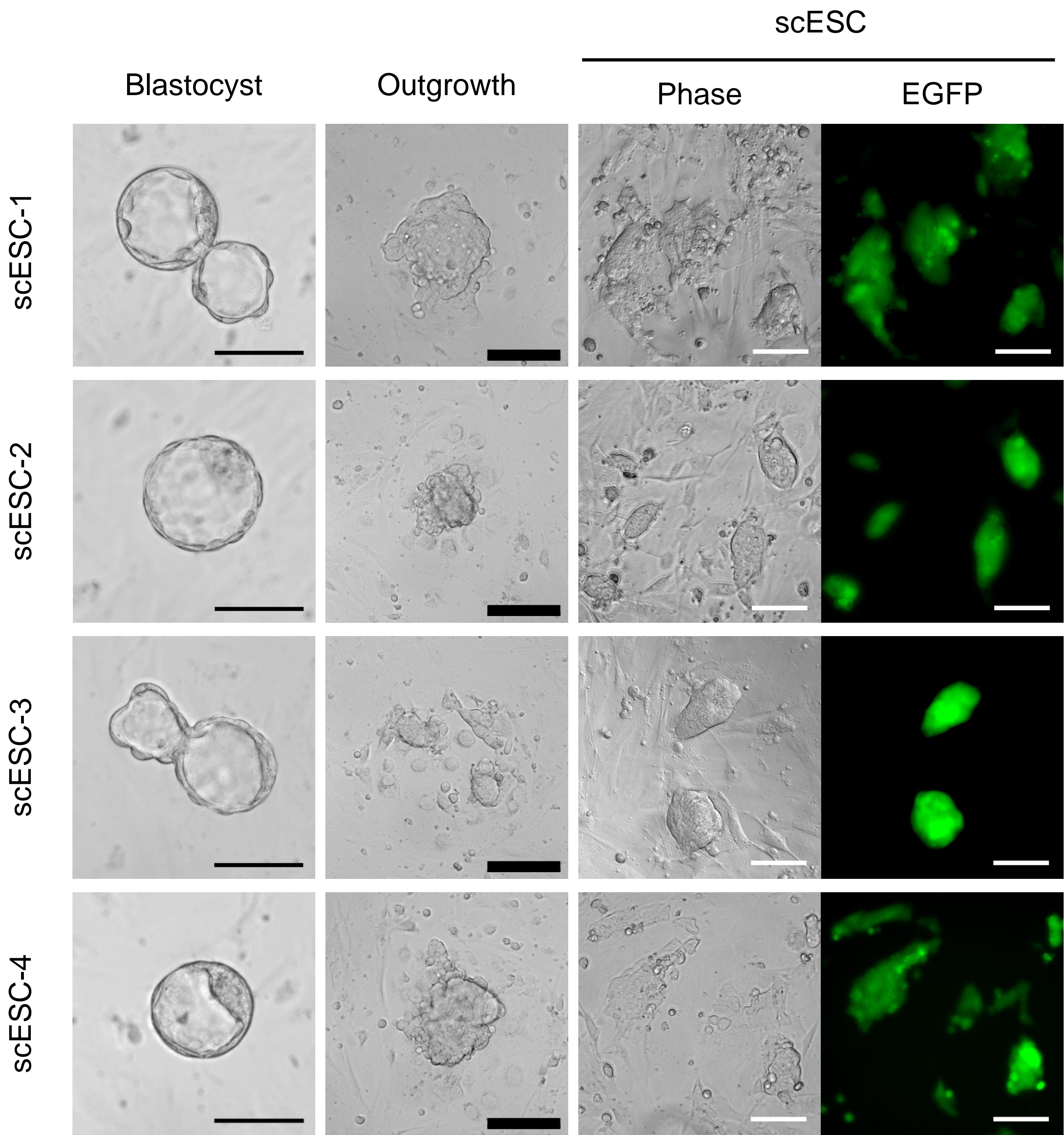

**B**

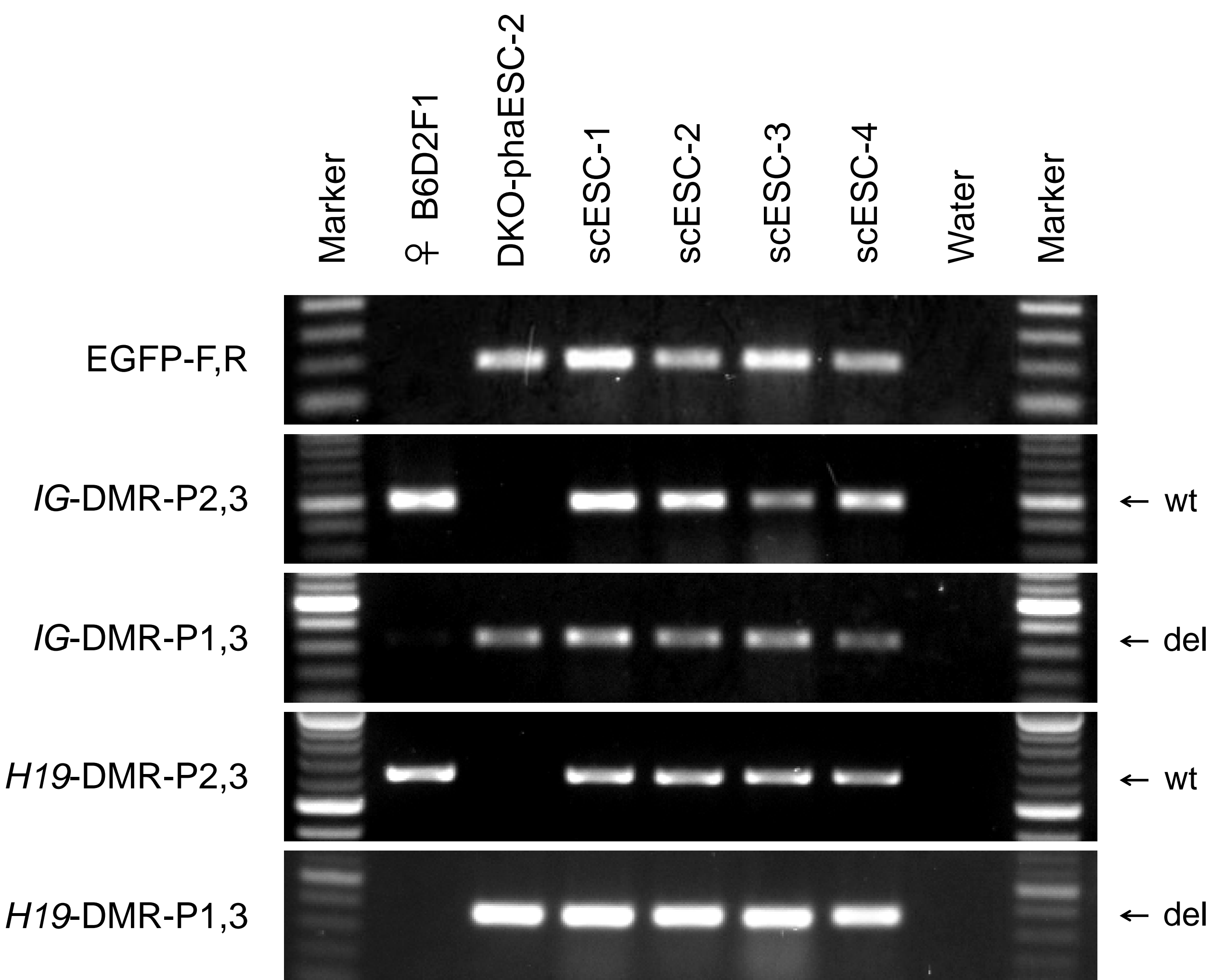

Supplement: S2 Fig — (A) Derivation of scESC lines from blastocysts generated by injection of DKO-phaESCs into oocytes. Images of blastocysts, outgrowth (passage 0) and scESCs after derivation are shown. Regular black bar, 100 μm; bold black bar, 200 μm; white bar, 100 μm. (B) Genotyping of 3 scESC lines. All 3 scESC lines exhibited both wild type and mutant alleles for the IG-DMR and H19-DMR, indicating both oocytes and DKO-phaESCs genome contributed to the genome of blastocysts. (PDF) [file pone.0233072.s002.pdf]

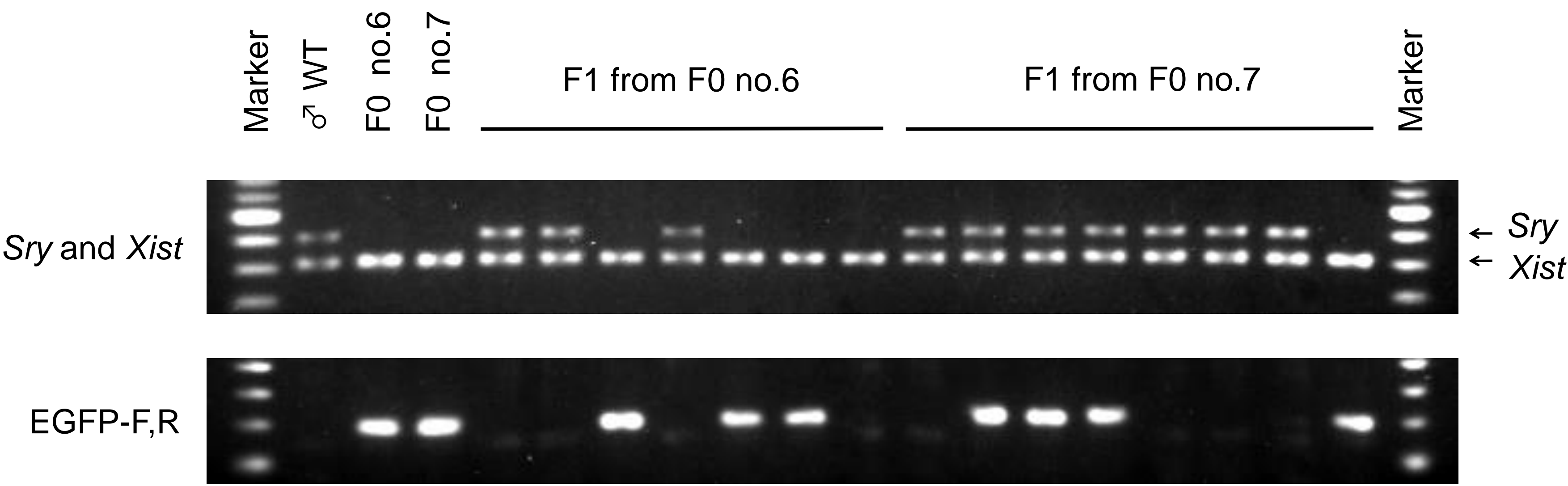

Supplement: S3 Fig — PCR-based genotyping was performed for 15 F1 mice born to semi-cloned females (F0 no.6 and 7) and wild type Swiss Webster males. EGFP transgene was inherited to 7 among 15 F1 mice. (PDF) [file pone.0233072.s003.pdf]
