# Supplementary material for: Polyploidy of semi-cloned embryos generated from parthenogenetic haploid embryonic stem cells
Source: PLoS One. 2020 Sep 10;15(9):e0233072. doi: 10.1371/journal.pone.0233072 (PMC7482839; doi:10.1371/journal.pone.0233072)
Supplement: S1 Table — (PDF) [file pone.0233072.s004.pdf]

S1 Table. List of oligos

Name	Sequence (5' to 3')	Application
EGFP-F	GAC GTA AAC GGC CAC AAG TTC	Genotyping
EGFP-R	GAA GAA GTC GTG CTG CTT CAT GTG	Genotyping
H19-DMR-P1	GTG GTT AGT TCT ATA TGG GG	Genotyping
H19-DMR-P2	AGA TGG GGT CAT TCT TTT CC	Genotyping
H19-DMR-P3	TCT TAC AGT CTG GTC TTG GT	Genotyping
IG-DMR-P1	TGT GCA GCA GCA AAG CTA AG	Genotyping
IG-DMR-P2	CCA CAA AAA CCT CCC TTT CA	Genotyping
IG-DMR-P3	ATA CGA TAC GGC AAC CAA CG	Genotyping
SRY-2	TCT TAA ACT CTG AAG AAG AGA C	Genotyping
SRY-4	GTC TTG CCT GTA TGT GAT GG	Genotyping
Xist-14	GTA GAT ATG GCT GTT GTC AC	Genotyping
Xist-16	CTC CAT CCA AGT TCT TTC TG	Genotyping
H19-DMR-gRNA1-F	CAC CCA TGA ACT CAG AAG AGA CTG	gRNA
H19-DMR-gRNA1-R	AAA CCA GTC TCT TCT GAG TTC ATG	gRNA
H19-DMR-gRNA2-F	CAC CAG GTG AGA ACC ACT GCT GAG	gRNA
H19-DMR-gRNA2-R	AAA CCT CAG CAG TGG TTC TCA CCT	gRNA
IG-DMR-gRNA1-F	CAC CCG TAC AGA GCT CCA TGG CAC	gRNA
IG-DMR-gRNA1-R	AAA CGT GCC ATG GAG CTC TGT ACG	gRNA
IG-DMR-gRNA2-F	CAC CCT GCT TAG AGG TAC TAC GCT	gRNA
IG-DMR-gRNA2-R	AAA CAG CGT AGT ACC TCT AAG CAG	gRNA
Tracr-Rev	AAA AAA AGC ACC GAC TCG GTG CC	gRNA cloning
U6-Fwd	GAG GGC CTA TTT CCC ATG ATT CC	gRNA cloning
Dlk1-F	ACT TGC GTG GAC CTG GAG AA	RT-PCR
Dlk1-R	CTG TTG GTT GCG GCT ACG AT	RT-PCR
Gapdh-F	AGG TCG GTG TGA ACG GAT TTG	RT-PCR
Gapdh-R	TGT AGA CCA TGT AGT TGA GGT CA	RT-PCR
Gtl2-F	TTG CAC ATT TCC TGT GGG AC	RT-PCR
Gtl2-R	AAG CAC CAT GAG CCA CTA GG	RT-PCR
H19-F	CAT GTC TGG GCC TTT GAA	RT-PCR
H19-R	TTG GCT CCA GGA TGA TGT	RT-PCR
Igf2-F	CTA AGA CTT GGA TCC CAG AAC C	RT-PCR
Igf2-R	GTT CTT CTC CTT GGG TTC TTT C	RT-PCR
Igf2r-bs-F	TAG ATA TTT TGG GGA ATT GAG G	Bisulfite PCR
Igf2r-bs-R	CCC CCC TCC CTT CTC CTC TTA CTA	Bisulfite PCR
Kcnq1-bs-F	AAG TTT GGG TTA TAA AGA TGG GG	Bisulfite PCR
Kcnq1-bs-R	CTC ATC ATA ACC TCC CCC TCC T	Bisulfite PCR
Peg13-bs-F	GGT CAT AGA GTT GTA GTA AAG GGG	Bisulfite PCR
Peg13-bs-R	CAA AAT ATA TCT CCA CCR AAC AAA TTA C	Bisulfite PCR
pJet1.2 fwd	CGA CTC ACT ATA GGG AG	Bisulfite sequencing
