# Supplementary material for: Polyploidy of semi-cloned embryos generated from parthenogenetic haploid embryonic stem cells
Source: PLoS One. 2020 Sep 10;15(9):e0233072. doi: 10.1371/journal.pone.0233072 (PMC7482839; doi:10.1371/journal.pone.0233072)
Supplement: Attachment — Submitted filename: Point_To_Point_Response.pdf [file pone.0233072.s006.pdf]

Point to point response to the comments for the authors

1. Lines 223-227.

The description of this Figure is partially incorrect, and the conclusion is still overstated:

- The sentence “patterns …………appear hypermethylated” is gramatically incorrect.

- The Peg13 locus in no.6 is not hypermethylated compared to control. It should be mentioned that the control is Female B6D2F1.

- It appears the Peg13 locus lost some methylation

- The fact that imprinting is mostly maintained, supports the idea that methylation patterns are “normal”

- Introduce “mostly” to tone down this conclusion: The 2 semi-cloned mice possessed mostly normal methylation patterns.

I would personally be curious regarding the methylation status of the oocyte-derived allele of th DMRs. Although not manipulated, showing that their methylation status is as predicted would have supported the “mostly” normal methylation patterns.

Response: We have corrected the sentence “patterns … appear hypermethylated” and changed the description of DNA methylation at the Peg13. The sentence reads now “Conversely, Peg13 was less methylated in F0 no. 6 and 7 than in the control”

We agree that an analysis of methylation at the H19- or IG-DMR would have allowed us to confirm the unmethylated status of the two DMRs on the oocyte derived chromosomes. Normal development of the two semi-cloned mice is consistent with the expected normal methylation at these 2 imprinted loci. Considering this point, we have toned down our conclusion. The section reads now “Considering that we observed slightly increased methylation levels in the control, which was comparable to that in F0 no. 6 and no. 7, we suggest that methylation of the 3 imprinted genes that we investigated was within a normal range in the 2 semi-cloned mice. This view is consistent with our observation that the semi-cloned mice were healthy and fertile.”

2. Lines 275-277

It is not clear to me what is being expressed in this sentence. Can that be rephrased?

Response: We have rephrased this paragraph. It is harder to explain how mixed diploid and tetraploid cells could have arisen in a single semi-cloned embryo and we have extended the discussion and include two additional references. We hope that the revised paragraph will be informative for the reader.
